# Carcinosarcoma of the Ureter with a Small Cell Component: Report of a Rare Pathologic Entity and Potential for Diagnostic Error on Biopsy

**DOI:** 10.1155/2014/391615

**Published:** 2014-12-24

**Authors:** Kent Newsom, Bayo Tojuola, Samer Al-Quran, Sijo Parekattil, William Hamilton, Lizette Vila Duckworth

**Affiliations:** ^1^Department of Pathology, Immunology and Laboratory Medicine, University of Florida College of Medicine, P.O. Box 100275, 1600 SW Archer Road, Gainesville, FL 32610-0275, USA; ^2^Department of Urology, University of Florida, Gainesville, FL 32610, USA; ^3^South Lake Hospital in Partnership with Orlando Health, 1900 Don Wickham Drive, Clermont, FL 34711, USA

## Abstract

Carcinosarcomas of the ureter are rare biphasic neoplasms, composed of both malignant epithelial (carcinomatous) and malignant mesenchymal (sarcomatous) components. Carcinosarcomas of the urinary tract are exceedingly rare. We report a unique case of a carcinosarcoma of the ureter with a chondrosarcoma and small cell tumor component arising in a 68-year-old male who presented with microscopic hematuria. CT intravenous pyelogram revealed right-sided hydroureter and hydronephrosis with thickening and narrowing of the right ureter. The patient underwent robot-assisted ureterectomy with bladder cuff excision and subsequent adjuvant chemotherapy. The patient is disease-free at 32 months after treatment. We provide a brief synoptic review of carcinosarcoma of the ureter and bladder with utilization of immunohistochemical (IHC) stains and potential diagnostic pitfalls.

## 1. Introduction

Carcinosarcomas of the ureter are rare malignant neoplasms with only around a dozen case reports within the literature [1–14]. They are biphasic neoplasms composed of both malignant epithelial and mesenchymal components. These aggressive tumors are more commonly found within the uterus, where they are also known within the gynecologic pathology nomenclature as malignant mixed mesodermal tumors (MMMT). They are usually present in elderly patients with a median age of 72 (mean age 64) and are histologically of high grade with an advanced stage at diagnosis and subsequent poor prognosis. Of reported cases of ureteral carcinosarcoma, the epithelial components most often are comprised of urothelial (transitional cell) carcinoma, carcinoma in situ, adenocarcinoma, squamous cell carcinoma, or small cell carcinoma. The stromal or sarcomatous component most often consists of osteosarcoma, chondrosarcoma, leiomyosarcoma, or rhabdomyosarcoma [13, 14]. Due to the limited number of cases, there is no clear consensus on treatment regimens and impact on survival. We present a rare case of carcinosarcoma of the ureter and review the histopathogenesis and immunohistochemical staining profile and discuss current treatment strategies and prognosis.

## 2. Case Presentation

**Figure 1:**
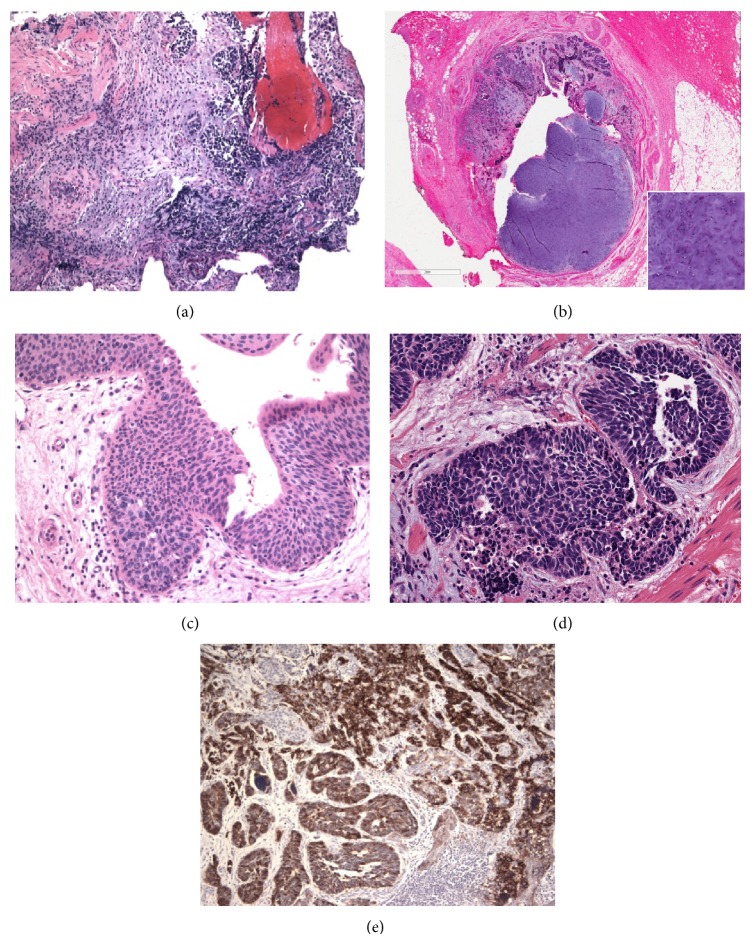
Histologic appearance of carcinosarcoma of the ureter. (a) Biopsy demonstrating poorly differentiated, dark blue cell tumor with marked crush artifact (5x, H&E). (b) Ureterectomy specimen demonstrating tumor obliterating the ureter lumen with high grade chondrosarcoma component (5x and insert 20x, H&E). (c) Ureter with urothelial carcinoma in situ (20x, H&E). (d, e) Small cell carcinoma component in carcinosarcoma showing strong and diffuse immunoreactivity with CD56 (e) (20x, H&E and 10x, IHC).

A 68-year-old male presented with microscopic hematuria on routine physical exam, and further work-up revealed right-sided hydronephrosis and hydroureter with a transition zone in the right ureter. The patient was referred to our institution for consideration of right-sided robot-assisted ureteral excision. Evaluation with ureteroscopy found a mass in the right mid to distal ureter that was biopsied and read as high grade urothelial carcinoma. The patient subsequently underwent robotic-assisted right distal ureterectomy with bladder cuff excision. Macroscopically, there was no distinct mass but an ulcerated and hemorrhagic mucosa in the right ureter extending 7 cm in length with concurrent stricture. Microscopic examination of this specimen revealed a biphasic neoplasm composed of both malignant epithelial and mesenchymal components (Figure 1). The epithelial components consisted of high grade urothelial carcinoma with multifocal carcinoma in situ, focal areas demonstrating glandular/enteric differentiation, and a small cell carcinoma component. The mesenchymal component was composed of a high grade chondrosarcoma.

Immunohistochemical studies demonstrated that the epithelial components were immunoreactive with CAM 5.2, cytokeratin 7, and focally with cytokeratin 20. The small cell carcinoma component was positive for CD56, but other neuroendocrine markers including synaptophysin and chromogranin were negative. The high grade chondrosarcoma component was not stained. The morphologic and immunoprofile was consistent with a carcinosarcoma. The tumor was muscle invasive and the pathologic stage was pT2NX, according to the AJCC 7th edition. The patient was given the option of adjuvant therapy secondary to the high grade nature of the malignacy with muscle invasion and concurrent small cell carcinoma component. He received three cycles of cisplatin and gemcitabine chemotherapy. After 32 months of follow-up, the patient is alive and disease-free with negative CT and PET scans.

## 3. Discussion

Carcinosarcomas are aggressive neoplasms that can occur anywhere in the body but commonly occur within the uterus in women and the bladder in men. These malignant tumors most often occur in older patients, greater than 60 years of age, and are more frequent in men than women (M : F ratio of 4 : 1) [13]. Carcinosarcomas of the ureter are biphasic tumors with distinct carcinoma and sarcomatous components. Historically within the literature there has been a lack of consistency and clarity in the histogenesis of these lesions as well as the nomenclature. The terms carcinosarcoma and sarcomatoid carcinoma (spindle cell neoplasms) have often been used interchangeably, although now we appreciate that these tumors are different based on the fact that carcinosarcomas have a true mesenchymal component distinctive from the epithelial component [15, 16]. Sarcomatoid carcinomas are epithelial-derived neoplasms that morphologically resemble sarcomas but only stain with epithelial markers such as cytokeratins (unlike carcinosarcomas which stain with both epithelial and mesenchymal markers). The origin and evolution of carcinosarcomas have also been questioned with multiple theories ranging from a common stem cell origin to a collision tumor resulting from two different tumors (sarcoma and carcinoma) converging within the same tumor [15, 16]. Recent studies have supported the monoclonality of these neoplasms, suggesting divergence from a common precursor stem cell origin [17].

Carcinosarcoma of the ureter is a rare malignant tumor with poor outcomes involving frequent recurrence within months and death from disease within two years [13, 14]. It is important to differentiate and distinguish carcinosarcomas from more common, and perhaps less aggressive, neoplasms that often occur in this region including carcinomas with osseous or cartilaginous metaplasia, spindle cell carcinomas (spindle cell neoplams), carcinomas with a pseudosarcomatous stroma, and collision tumors. This can be reliably done by the pathologist using immunohistochemical stains, cytological features, and relationship of the carcinoma to the stroma or suspected sarcomatous component of the tumor. The epithelial components of ureteral carcinosarcomas often include urothelial carcinoma, carcinoma in situ, small cell carcinoma, adenocarcinoma, and squamous cell carcinoma. The stromal components often consist of chondrosarcoma, osteosarcoma, leiomyosarcoma, malignant fibrous histiocytoma, and, less commonly, rhabdomyosarcoma. Potential pitfalls confronting pathologists can distinguish a high grade urothelial carcinoma from a small cell/neuroendocrine neoplasm on biopsy alone, especially if there is tissue crush artifact or sampling bias. In these examples one might employ a neuroendocrine marker, in addition to a cytokeratin, to rule out small cell carcinoma, as this diagnosis could affect preoperative or postoperative oncologic therapy and prognosis. Prior literature indicates that CD56 is the immunohistochemical stain that most consistently demonstrates positive staining in small cell tumors [18]. Most patients with carcinosarcoma of the ureter present clinically with hematuria (microscopic and gross) and evidence of ureteral obstruction with obstructive nephropathy. Imaging can often locate a space occupying lesion of the ureter or retroperitoneum. Significant past medical or social history can include exposure to chemotherapy or radiation for risk of developing carcinosarcoma in general. Smoking history has been identified as a possible significant contributing factor for rare primary small cell carcinomas of the ureter [18]. Our patient with a small cell tumor component had a past medical and social history significant for smoking.

Aggressive surgical management is the treatment of choice in management of patients with carcinosarcoma of the ureter. Literature reports suggest that nephroureterectomy is the recommended procedure for surgically treating these lesions [13]. This potentially depends on the focality of the tumor and proximity of the tumor to the kidney. In our patient, the location of the tumor in the mid to distal ureter lent itself to a minimally invasive approach given the patient's age and medical comorbidities. A strong need to preserve the patient's renal function was an added influence in decision making. A robotic-assisted laproscopic resection of the diseased segment of ureter and bladder cuff was performed with ureteroneocystostomy. Though the patient had high grade disease on biopsy, a thorough ureteroscopy was performed to ensure that there was no disease in the rest of the ureter and renal pelvis. Biopsies of the disease-free margin of ureter were taken and were negative. Renal pelvis washings were also performed and revealed no malignant cells.

Secondary to the rarity of these lesions, low number of patients to conduct meaningful prospective studies, and lack of long term follow-up, there is no clear consensus on radiochemotherapy regimens and impact on survival. The literature suggests that the prognosis is universally poor and that neoadjuvant or adjuvant chemotherapy and radiotherapy may not improve prognosis [13, 14]. However, reports involving carcinosarcoma of the bladder, which is far more common than carcinosarcoma of the ureter, have demonstrated that a small number of patients have experienced a survival benefit with combined surgery and radiation or chemotherapy [19–21], with one case obtaining a complete pathologic response after neoadjuvant chemoradiotherapy [19]. The studies directly correlate stage of tumor with survival and disease-free interval [20, 21]. Additionally, studies involving rare, pure small cell carcinomas of the ureter have demonstrated a small survival advantage in patients who underwent surgical resection and adjuvant chemotherapy, which is pertinent in cases of carcinosarcoma with a small cell component [18].

Given the concern for residual microscopic disease and final pathology in our case, the decision was made to proceed with adjuvant chemotherapy. A combination of cisplatin and gemcitabine was used. The organ of disease origin and type of epithelial component in carcinosarcomas may be important factors in deciding upon which regimen to use. In addition, some confounding variables which could account for the apparent ineffectiveness of chemotherapy regimens include improper histological classification, focality of the tumor, size and depth of invasion, distance from or involvement of the renal pelvis, and/or genetic makeup of the tumor itself (inherently aggressive and unresponsive to treatment). Our patient is currently disease-free 32 months after treatment, suggesting a role for combined surgical and oncological treatment in the management of these patients.

Recognition of this rare entity is important for urologists and pathologists alike to accurately diagnose these lesions and treat them aggressively. Though overall prognosis remains poor for the majority of patients, our case suggests multimodal therapy may provide an avenue for prolonged survival in carcinosarcomas of the ureter.
